# Successful Pain Relief With Duloxetine in a Patient With Chronic Postsurgical Pain After Open Reduction and Internal Fixation: A Case Report

**DOI:** 10.7759/cureus.20456

**Published:** 2021-12-16

**Authors:** Takahiro Machida, Hinako Katayama, Osamu Yoshida, Akihisa Watanabe

**Affiliations:** 1 Orthopaedics, Machida Orthopaedics, Kochi, JPN; 2 Rehabilitation, Machida Orthopaedics, Kochi, JPN

**Keywords:** proximal humeral fracture, open reduction and internal fixation, duloxetine, conditioned pain modulation, chronic postsurgical pain

## Abstract

Chronic postsurgical pain (CPSP) is a common complication of surgery. We report that a patient with CPSP after open reduction and internal fixation (ORIF) had pain relief with duloxetine, and that the conditioned pain modulation (CPM) efficiency may predict the efficacy of duloxetine. A 54-year-old woman with CPSP after ORIF due to proximal humeral fracture was presented to our orthopedic clinic one month after surgery. Despite several analgesics, she still had pain three months after surgery, pain during activity was 74 on the visual analogue scale (VAS), 16 on the American Shoulder and Elbow Surgeons Standardized Shoulder Assessment Form (ASES), 18 on the PainDETECT questionnaire, and CPM efficiency was -5.7%. The patient was treated with duloxetine, starting at 20mg/day and increasing every week. Three months after starting duloxetine, pain on the VAS was 18, ASES was 61, PainDETECT questionnaire was 6, and CPM efficiency was -39.8%. The dose of duloxetine was decreased every week and then withdrawn. Neuropathic pain may be involved even in patients with CPSP after ORIF, and duloxetine may be efficacious in such cases. CPM testing may provide useful information for clinicians in selecting appropriate drugs and in determining when to withdraw drugs.

## Introduction

Chronic postsurgical pain (CPSP), defined as moderate to severe pain lasting three months after surgery [1], is a common complication associated with surgery. The causes of CPSP are believed to be multiple, including neuropathic pain (caused by a lesion or disease of the somatosensory nervous system) [2], and persistent muscle or ligament spasm and pain (as a result of a severe inflammatory reaction following surgery) [3]. Many studies of CPSP in orthopedic surgery including total knee arthroplasty and spine surgery have reported that some patients develop neuropathic pain [4,5]. However, there is limited evidence after open reduction and internal fixation (ORIF) for fractures.

Duloxetine is one of the most efficacious drugs for neuropathic pain. Recent studies reported that duloxetine was efficacious in CPSP patients who underwent total knee arthroplasty and spine surgery [5,6]. Furthermore, a study in patients with painful diabetic neuropathy showed that the baseline conditioned pain modulation (CPM) efficiency predicted the efficacy of duloxetine [7]. However, it is unclear whether CPM efficiency also predicts the efficacy of duloxetine in CPSP patients.

Here, we report the short-term results of a patient with CPSP after ORIF due to proximal humeral fracture. The patient had pain relief with duloxetine, and the CPM testing may provide useful information for understanding the pathophysiology of pain and drug selection in patients.

## Case presentation

**Figure 1:**
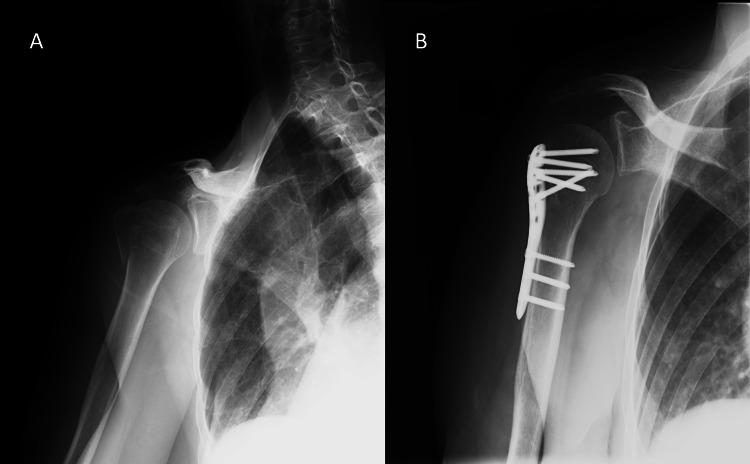
X-ray of the right shoulder A) before surgery B) at one month after surgery

A 54-year-old woman with a history of proximal humeral fracture of the right side due to fall (Neer classification: 2-part greater tuberosity; AO classification: 11-A1), who underwent ORIF, was referred to our orthopedic clinic one month after surgery (Figure 1A, 1B). According to the post-surgery protocol of the referring hospital, elevation of the shoulder joint was prohibited until four weeks after surgery, and rehabilitation for range-of-motion exercises was to be started on the fifth week after surgery.

**Figure 2:**
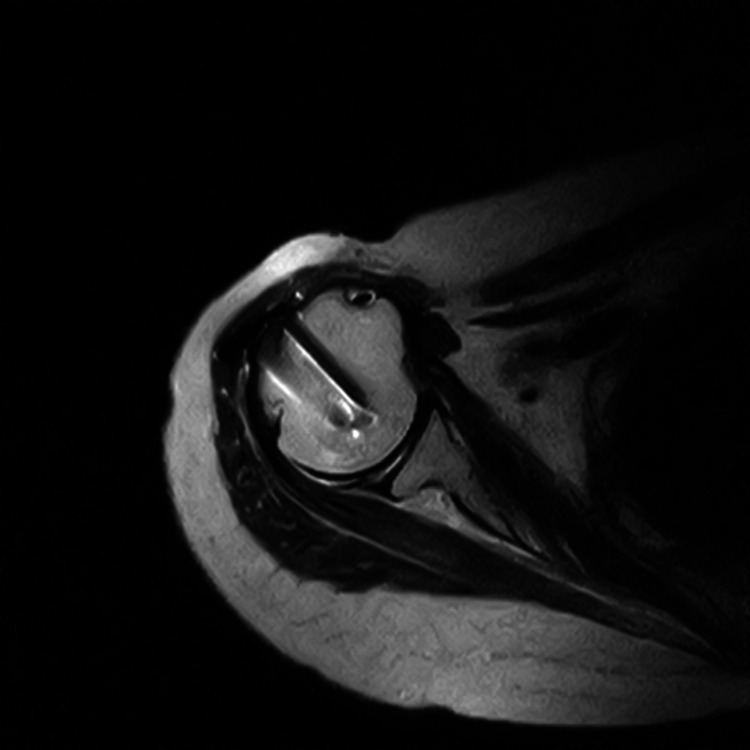
Axial T1-weighted magnetic resonance imaging Magnetic resonance imaging at three months after surgery. The greater tuberosity of the humerus has healed.

Despite one corticosteroid injection and several analgesics (tramadol hydrochloride 100 mg/day, celecoxib 200 mg/day), she was still in pain three months after surgery and had not made adequate progress in rehabilitation.Upon examination at three months after surgery, pain during activity was 74 on the visual analogue scale (VAS: 0 = no pain, 100 = worst pain imaginable), 16 on the American Shoulder and Elbow Surgeons Standardized Shoulder Assessment Form (ASES), and 82 on the Shoulder Pain and Disability Index (SPADI) (Table 1). Magnetic resonance imaging confirmed the union of the greater tuberosity of the humerus (Figure 2). The cervical spine and rotator cuff were carefully examined, but no abnormalities were found. She had no other pain-related conditions including complex regional pain syndrome, cervical spondylosis, inflammatory arthritis, adhesive arthritis, diabetes, thyroid disease, infections, or psychiatric disorders such as depression. She had no previous history of chronic pain.

**Table 1:** Course of pain, function, and conditioned pain modulation efficiency VAS, visual analogue scale; ASES, American Shoulder and Elbow Surgeons Shoulder Score; SPADI, Shoulder Pain and Disability Index; DN-4, Douleur Neuropathique 4; PD-Q, PainDETECT questionnaire; CPM, conditioned pain modulation; n/a, not available CPM efficiency is defined as follows:* (pre_PPT – post_PPT) / pre_PPT* [13]

	After surgery, months							
	1	2	3	4	5	6	7	12
Pain during activity, VAS	95	95	74	15	10	18	12	14
Range of motion, passive elevation	70	95	130	130	130	160	160	160
, active elevation	10	10	30	30	90	90	130	160
ASES	3	3	16	54	69	61	72	81
SPADI	97	95	82	72	65	18	18	16
DN-4	n/a	n/a	7	n/a	n/a	3	n/a	3
PD-Q	n/a	n/a	18	n/a	n/a	6	n/a	6
CPM efficiency, %	n/a	n/a	-5.7	n/a	n/a	-39.8	n/a	n/a

We conducted a specific evaluation of central nervous system dysfunction and neuropathic pain. Scores on the Douleur Neuropathique 4 (DN-4) was 7, and PainDETECT questionnaire (PD-Q) was 18. CPM efficiency (see below) was -5.7% in the right forearm. Based on the above, the involvement of neuropathic pain and dysfunction of the endogenous pain inhibitory pathway was considered, and administration of duloxetine was started.

The dosing protocol for duloxetine was as follows: the initial dose was 20mg/day for a week, then 40mg/day for a week, and finally 60mg/day [8], with constant assessment of adverse events. The dosage should be maintained until satisfactory pain relief is obtained. When satisfactory pain relief is obtained, or when adverse events are reported, the dose should be gradually decreased on a week-by-week basis.

Within one month of starting duloxetine, pain improved rapidly to 15 on the VAS. Rehabilitation, which had been ongoing throughout the postoperative period, was continued appropriately thereafter. Three months after starting duloxetine, CPM efficiency was -39.8%, the screening for neuropathic pain were DN-4 of 3 and PD-Q of 6, suggesting an improvement in CPM efficiency. Therefore, the dose of duloxetine was decreased every week and withdrawn after four months (seven months after surgery). Pain and function improved, with VAS of 12, ASES of 71, and SPADI of 18 (Table 1).

Adverse events were reported during this medication period: drowsiness occurred one week after increasing the dose of duloxetine to 60mg/day. The dosage was promptly decreased to 40mg/day. This adverse event was resolved rapidly with the dose decrease.

CPM testing procedure

The CPM testing procedure followed a standardized CPM protocol for clinical studies [9], “conditioning stimulus” with cold water [10], and “test stimulus” with mechanical pressure [11], which has been reported to be the most reproducible and useful method [12]. First, mechanical pressure was evaluated using a digital pressure algometer (Standard Model Digital Force Gauge ZTS series, IMADA CO., LTD., Aichi, Japan). The pressure pain threshold (PPT) was calculated by averaging two measurements on the right side of the forearm. The mechanical force was applied using a 1.0 cm2 probe, with the pressure increased at 30kPA/s. Next, responses to cold water were measured. The left hand was immersed in cold water (10°C) for 60s, the pain level was set at 40/100 or higher. PPT was measured in the same way, while the patient kept her hand in the water. CPM efficiency was defined as the percentage change:

CPM efficiency =* (pre_PPT - post_PPT) / pre_PPT* [13]: Negative values indicated an efficient CPM.

The examination time for CPM testing was less than 10 minutes. All procedures were performed by one observer with more than 10 years of experience.

## Discussion

The most important findings from this patient were that neuropathic pain may be involved even in patients with CPSP after ORIF, in which case duloxetine may be efficacious, and CPM efficiency may predict the efficacy of duloxetine even in patients after ORIF.

This patient had neuropathic pain. Although the bony healing was confirmed by magnetic resonance imaging three months after surgery, the pain persisted. We hypothesized that the pain was not due to delayed fracture healing but due to neuropathic pain, which was confirmed by DN-4 and PD-Q scores. Neuropathic pain can also be involved in osteoarthritis of the knee [4] and chronic non-specific low back pain [5], which were previously thought to be nociceptive-type pains. Furthermore, the patient had successful pain relief with duloxetine, even though other analgesics had failed to relieve her pain. Duloxetine is commonly used for fibromyalgia and chronic musculoskeletal pain as well as diabetic neuropathy [11]. Furthermore, duloxetine administration for CPSP has been investigated because neuropathic pain may be involved in total knee arthroplasty and spine surgery [14,15]. In this case report, duloxetine was also efficacious in a patient with CPSP after ORIF due to proximal humeral fracture in which neuropathic pain was involved.

The second clinically important finding is that CPM efficiency may predict the efficacy of duloxetine even in patients after ORIF. CPM testing could help develop personalized pain management strategies based on an individual’s endogenous analgesic capacity [12], and inefficient baseline CPM was expected to benefit from duloxetine in diabetic neuropathy [7]. In a study aimed at determining a reference value of CPM efficiency, the mean CPM efficiency was reported to be -27.5% [16]. In contrast, this patient had an inefficient baseline CPM of -5.7% (at three months after surgery) and thus, was expected to benefit from duloxetine. In fact, three months of duloxetine treatment was efficacious with improved pain and improved CPM efficiency. Similar findings have shown that baseline CPM efficiency predicts the efficacy of the drug pregabalin for chronic pancreatitis [17] and tapentadol for diabetic polyneuropathy [18]. Additionally, baseline CPM efficiency has been shown to affect the efficacy of pregabalin even in healthy volunteers [19].

This patient had success with duloxetine and rehabilitation, not only in terms of pain but also in terms of function. Functional recovery had lagged pain relief by a few months, which suggests that successful pain management led to successful rehabilitation. Appropriate management of the CPSP may have been an effective step toward successful rehabilitation. Situations where rehabilitation is delayed due to the CPSP should be avoided. Proper management of CPSP is believed to improve clinical success and reduce disability [20].

The course of this patient provides useful insights for clinicians: if pain persists after ORIF, neuropathic pain may be involved and should be assessed with DN-4 or PD-Q. Pain relief for neuropathic pain should be expected with the use of appropriate drugs, and clinicians should be aware that they may have unfortunately overlooked patients after ORIF who would benefit from analgesic treatment with duloxetine. CPM testing may provide useful information for clinicians in selecting appropriate drugs and in determining when to withdraw drugs.

## Conclusions

In conclusion, pain relief in this patient was multifactorial, with duloxetine contributing to one of the factors. Even in patients after ORIF, duloxetine may be efficacious for neuropathic pain, and CPM efficiency may predict the efficacy of duloxetine. This case report suggests that the CPM testing may provide important information for drug selection in patients after ORIF and may help clinicians make decisions in the process of finding the optimal drug for each individual patient.
